# Geopropolis from *Melipona fasciculata* Smith Accelerates Wound Healing in Diabetic Mice

**DOI:** 10.3390/metabo15060413

**Published:** 2025-06-19

**Authors:** Aramys Silva Reis, Gabriel Carvalho de Souza, Guilherme Martins Gomes Fontoura, Luecya Alves de Carvalho Silva, Alberto Jorge Oliveira Lopes, Richard Pereira Dutra, Lucilene Amorim Silva, Rosane Nassar Meireles Guerra, Maria Nilce Sousa Ribeiro, Flávia Raquel Fernandes Nascimento

**Affiliations:** 1 Programa de Pós-Graduação em Saúde e Tecnologia, de Ciências de Imperatriz, Universidade Federal do Maranhão, Imperatriz 65915-240, Maranhão, Brazil; guilherme.fontoura@discente.ufma.br (G.M.G.F.); richard.dutra@ufma.br (R.P.D.); 2 Laboratório de Fisiopatologia e Investigação Terapêutica, Curso de Medicina, Centro de Ciências de Imperatriz, Universidade Federal do Maranhão, Imperatriz 65915-240, Maranhão, Brazil; gabriel.medufma@gmail.com (G.C.d.S.); luecya.carvalho@ufma.br (L.A.d.C.S.); 3 Programa de Pós-Graduação em Química, Instituto Federal de Educação, Ciência e Tecnologia do Maranhão, São Luís 65030-005, Maranhão, Brazil; lopesajo@gmail.com; 4 Laboratório de Química de Produtos Naturais, Curso de Licenciatura em Ciências Naturais, Centro de Ciências de Imperatriz, Universidade Federal do Maranhão, Imperatriz 65915-240, Maranhão, Brazil; 5 Programa de Pós-Graduação em Ciências da Saúde, Centro de Ciências Biológicas e da Saúde, Universidade Federal do Maranhão, São Luís 65085-580, Maranhão, Brazil; lucilene.silva@ufma.br (L.A.S.); rosane.guerra@ufma.br (R.N.M.G.); maria.nilce@ufma.br (M.N.S.R.); 6 Laboratório de Patologia e Imunoparasitologia, Universidade Federal do Maranhão, São Luís 65085-580, Maranhão, Brazil; 7 Laboratório de Imunofisiologia, Universidade Federal do Maranhão, São Luís 65085-580, Maranhão, Brazil; 8 Departamento de Farmácia, Centro de Ciências Biológicas e da Saúde, Universidade Federal do Maranhão, São Luís 65085-580, Maranhão, Brazil

**Keywords:** chronic wound healing, natural product, bioproduct

## Abstract

Background: Diabetic foot ulcers present a significant clinical challenge because of their high prevalence and severe complications. The need for innovative and accessible treatment options is critical. Owing to their medicinal properties, natural products, such as geopropolis, hold promise. However, the wound healing potential of the geopropolis of *Melipona fasciculata*, particularly in accelerating the healing of diabetic ulcers, remains unexplored. In this study, we evaluated the ability of the geopropolis of *M. fasciculata* to promote wound healing in diabetic mice. Methods: Geopropolis was collected, prepared as a hydroalcoholic extract, and formulated into a topical cream. Non-obese diabetic (NOD) mice with induced chronic wounds were treated with this cream daily, and wound healing was assessed through macroscopic measurements, histological analysis, cytokine quantification, and in silico molecular docking studies. Results: The results demonstrated that, compared with the control treatment, the geopropolis cream accelerated wound closure at all the analyzed time points (days 3, 7, and 14), reduced inflammatory infiltrates, and enhanced fibroblast proliferation and collagen deposition. These alterations were particularly pronounced in the final phase of healing, indicating an improvement in wound repair processes. These effects occurred without altering systemic cytokine levels, suggesting a localized treatment action. These results may be partially associated with the theoretical ability of beta-amyrin and cycloartenol to interact with human myeloperoxidase (MPO), as suggested by in silico docking analysis. Conclusions: Overall, the findings indicate that geopropolis cream could represent a viable alternative for managing diabetic ulcers, providing an effective means to enhance wound healing while remaining accessible to low-income populations.

## 1. Introduction

Diabetic foot ulcers (DFUs) are among the most severe and significant complications of diabetes mellitus (DM), affecting approximately 15–25% of individuals with this condition [1]. Each year, approximately 18.6 million people worldwide suffer from diabetic foot ulcers, with a five-year mortality rate of about 30% and a high risk of lower limb amputations, increasing this mortality rate to 70% [2].

Factors such as neuropathy and ischemia from peripheral arterial disease contribute to the development of diabetic foot lesions and impair their healing. Additionally, mechanical foot deformities lead to metabolic alterations that result in persistent inflammation. This scenario increases the chances of infection and leads to deficiencies in growth factors and the extracellular matrix, both of which are crucial for healing [3].

In developing countries, treating complex diabetic foot ulceration costs the equivalent of 5.7 years of annual income [4]. Consequently, specific products for treating this condition are expensive and inaccessible to low-income populations.

Natural products, such as propolis, could provide a more economical alternative for wound healing. Propolis is a resinous and balsamic substance obtained from various plants and enriched with wax and bee mandibular secretions [5]. However, most studies on the therapeutic properties of propolis have focused on bees of the *Apis* genus [6], with limited information available on the biological activities of propolis from stingless bees, such as those produced by bees of the genus *Melipona*.

In tropical and neotropical regions, particularly in South America, *Melipona fasciculata* species can be found [7,8]. These bees produce geopropolis from plant resinous materials enriched with bee mandibular secretions and soil addition [9]. The chemical composition of geopropolis varies depending on the local flora and geographic region [10,11], and it commonly contains phenolics, triterpenes, steroids, alcohols, sugars, organic acids, and fatty acids. These substances have been identified in chemical characterization studies of the same sample used in this work [11].

Although no studies have evaluated the healing effects of *M. fasciculata* geopropolis in chronic wounds, previous studies have demonstrated that this geopropolis has antimicrobial [12], immunomodulatory [13] and antioxidant [10,11,13] properties. These activities are relevant to the pathophysiology of diabetic wounds, which are characterized by persistent inflammation, oxidative stress, and impaired fibroblast activity. Additionally, Sousa-Fontoura et al. (2020) [14] demonstrated the healing potential of *Melipona subnitida* geopropolis in an acute wound model, reinforcing the relevance of geopropolis from stingless bees. These findings suggest that *M. fasciculata* geopropolis may contribute to the modulation of key processes involved in tissue repair under diabetic conditions, supporting its evaluation in a chronic wound model.

This study evaluated the healing potential of *M. fasciculata* geopropolis in non-obese diabetic (NOD) mice with induced chronic wounds. Diabetic mice are frequently used as models for chronic wound healing because of their similarity to the healing complications observed in human patients with diabetes mellitus.

## 2. Materials and Methods

### 2.1. Collection of Melipona fasciculata Smith Geopropolis Samples

For this study, geopropolis samples were collected from bees of the species *M. fasciculata* Smith from a meliponary located in the municipality of Palmeirândia, State of Maranhão, Brazil (2°40′80.3″ S and 44°52′66.1″ W). The municipality is in the flooded region of the Maranhao Lowlands (Baixada Maranhense). This region comprises various ecosystems, including mangroves, flood fields, lagoons, babassu fields, and forests [15].

To avoid external contamination, the samples were obtained directly from the beehives via a sterile spatula and refrigerated until extract preparation.

### 2.2. Hydroalcoholic Extract of Geopropolis (HEG) Preparation

The geopropolis sample (500 g) was macerated in a 1:2 (*w*/*v*) ratio with 70% ethanol for 48 h and filtered to separate the inorganic part (soil). The hydroalcoholic extractive solution (HEG) was concentrated in a rotary evaporator (Q344B2, Quimis, São Paulo, Brazil) and dried to a constant weight. The HEG was stored in amber flasks and refrigerated at 15 °C [10,11].

Prior chemical characterization of the HEG, coded as HEG2, had already been conducted by our group via gas chromatography-mass spectrometry (GC–MS). In this sample, triterpenoids, including cycloartenol (4.12%), beta-amyrin (2.66%), cycloursane (0.83%), and 3-oxo-urs-12-en-24-oic acid (0.61%), were the main compounds. Additionally, phenolic acids such as protocatechuic acid (0.38%) and gallic acid (1.03%) were identified, along with the steroid lanosterol (0.47%), as well as organic acids, sugars, and alcohols [11].

### 2.3. Geopropolis Cream Preparation

A hydrophilic anionic cream was prepared via the traditional phase inversion method [16] to apply HEG to the lesions. The HEG was then incorporated into the formulation at 2.5% (GF2.5) and 5% (GF5). All procedures were performed at room temperature in an environment free from impurities. Self-emulsifying Lanette N^®^ wax (Facial Pharmacy, São Luis, Brazil) at a concentration of 9% was used to prepare this formulation. Finally, microbiological control of the formulations was carried out, and no contamination was detected.

### 2.4. Animals

Non-obese diabetic (NOD) mice, which were 120 days old and weighed 25–35 g, were supplied by the Central Animal Facilities of the Federal University of Maranhão (UFMA) and maintained in conventional housing with a constant light–dark cycle (12:12 h) at the Animal Facility of the Laboratory of Immunophysiology (UFMA). The mice received water and regular diet food ad libitum. The animal study protocol was approved by the Ethics Committee of the Federal University of Maranhão (UFMA) (Protocol CEP/UFMA no. 23115.009717/2015-10) and was approved on 21 November 2016. The animal use and care guidelines were based on the standards established by the National Council for Control of Animal Experimentation (CONCEA).

NOD mice spontaneously develop autoimmune diabetes due to the progressive destruction of insulin-producing β-cells, with hyperglycemia typically manifesting between 10 and 12 weeks of age, especially in females [17,18]. Because it resembles human disease, this model is used to induce chronic lesions and is commonly employed to study diabetes-associated impairments in tissue repair [19].

Although the NOD model does not reproduce all aspects of human chronic wounds, such as ischemia or infection, it remains a relevant and validated approach for investigating hyperglycemia-related delays in wound healing. Its use is appropriate for evaluating topical interventions targeting inflammation and tissue regeneration under diabetic conditions.

### 2.5. Wound Model

To induce the wound, NOD mice with diabetes [blood glucose levels greater than or equal to 150 mg/dL (8.3 mmol/L)] were anesthetized with a 2:1 solution of xylazine hydrochloride (Rompum^®^, Bayer S.A., São Paulo, Brasil) (20 mg/kg) and 5% ketamine hydrochloride (Vetanarcol^®^, König S.A., Buenos Aires, Argentina) (25 mg/kg). The dorsal area of each animal was subsequently shaved and aseptically cleaned with 70% alcohol. After the area to be excised on the back of the animal was marked via a circular mold with a 1 cm diameter, the skin was removed with sterile forceps and scissors, creating a full-thickness excisional wound that included complete removal of the epidermis and dermis down to the subcutaneous tissue. Histological sections obtained on day 3 confirmed the absence of epithelial and dermal structures, validating the wound depth. Hemostasis was then achieved with digital pressure via sterile gauze soaked in saline solution for 1 to 2 min. After wounding, the animals were randomly assigned to four groups of twelve via a computer-generated sequence; an independent researcher labeled the cages to ensure allocation concealment. Animals showing signs of infection or accidental wound closure before day 3 were excluded; however, no animals or data points were excluded after allocation. Each group received a different topical treatment daily until tissue repair was complete. The negative control (NC group) was treated with 0.1 mL of Lanette cream. The positive control (PC group) received cream containing deoxyribonuclease (666 U/g), fibrinolysin (1 U/g), and chloramphenicol (10 mg/g) (Fibrase^®^ (Pfizer., São Paulo, Brasil) (DFC group) [20]. The experimental groups were treated with 2.5% (GF2.5 group) or 5% (GF5 group) geopropolis cream [21]. The experimental unit was one animal (one wound per mouse). The animals were monitored daily for general health and wound conditions. Humane endpoints included weight loss >15%, signs of severe pain (e.g., piloerection, hunched posture, reduced mobility), wound infection, or self-mutilation. No animals reached the humane endpoints during the study. The creams were prepared in a sterile environment. Before treating the lesions, the wounds were cleaned with 2 mL of sterile saline and then dried with clean gauze.

### 2.6. Macroscopic and Microscopic Evaluation

On days 0, 3, 7, and 14, the largest diameter (D) and smallest diameter (d) of the lesions were measured via a digital caliper. The area of the lesion was calculated via the formula for the area of a circle: A = (MD/2)^2^ × π, where MD is the arithmetic mean of D and d.

Four animals from each group were sacrificed on days 3, 7, and 14 for histopathological analysis. The lesions were excised, fixed in 10% formalin, and embedded in paraffin. Sections 5 µm thick were obtained and stained with hematoxylin–eosin (H&E) and Masson’s trichrome. The slides were analyzed under a common light optical microscope with 10× and 40× objectives. The following parameters were evaluated: inflammatory infiltration, edema, angiogenesis, fibroblastic proliferation, and collagen fibers. Tissue changes were scored as follows: 0 for absent, 1 for scarce, 2 for moderate, and 3 for intense. Measurements and histology scoring were performed by investigators who were blinded to the treatment groups.

### 2.7. Cytokine Assay

The serum concentrations of TGF-beta and IL-6 were quantified via enzyme-linked immunosorbent assay (ELISA) according to the manufacturer’s instructions (eBioscience, San Diego, CA, USA). For the assay, 100 µL of each supernatant was used. All the assays were performed in 96-well plates (Costa^®^, Corning, Lowell, MA, USA). The conversion of the absorbance values to concentrations was performed via linear regression from a standard curve obtained with known concentrations of recombinant cytokines.

### 2.8. In Silico Assay

#### 2.8.1. Ligand and Target Preparations

The molecular structures of silybin and the standard drugs were modeled and visualized via three-dimensional (3D) representations in GaussView 5.0.8 (Gaussian, Inc., Wallingford, CT, USA) [22]. The geometric and vibrational properties of these structures were computed in vacuo via density functional theory (DFT), specifically employing the B3LYP hybrid functional in conjunction with the 6-31++G(d,p) basis set, as implemented in Gaussian 16 [23].

The three-dimensional (3D) structures of human myeloperoxidase were obtained from the Protein Data Bank (PDB) under entry 6BMT. This structure was resolved using X-ray crystallography and exhibited acceptable structural quality parameters. For the purposes of analysis, all nonessential molecules present in the crystal structures were excluded, with a focus exclusively on a single residue within the active site.

#### 2.8.2. Molecular Docking

The molecular docking procedures were performed via AutoDock Vina 1.2.5 (The Scripps Research Institute, La Jolla, CA, USA) [24]. The structures of the selected targets and ligands for molecular docking (MD) were prepared via AutoDock Tools (ADT) version 1.5.7 [25]. In these simulations, the target structures were treated as rigid, while flexibility was allowed for each ligand. Gasteiger partial charges were calculated after the addition of hydrogens to both the ligands and target structures. The dimensions of the cubic grid box were set to 30 × 30 × 30 Å along the X, Y, and Z axes, centered on the selected active site residue Arg405. The number of docking modes was set to 80, with an exhaustiveness level of 80 [26]. The conformations with the most favorable interaction energies for the ligand–receptor complexes were selected on the basis of binding free energy, visual inspection, and analysis of residues involved in ligand interactions. Molecular analysis and visualization of the complexes were carried out via the UCSF Chimera package [27] and PoseView [28].

Furthermore, redocking experiments were performed on the PDB structures with their native ligands, following the same conditions and protocols applied to the newly identified compounds. The root mean square deviation (RMSD) was used to evaluate the predicted ligand positions from the docking simulations relative to their original positions in the crystal structures. This evaluation was carried out via the Swiss PDB Viewer. The RMSD assessment was critical in validating the accuracy and reliability of the docking methodology.

### 2.9. Statistical Analysis

Statistical analyses were performed via GraphPad version 8.0 software. Data normality was assessed via the Shapiro–Wilk test. The results are expressed as the means ± standard deviations for groups of 12, 8, or 4 animals. When the data were parametric, comparisons were made via two-way ANOVA; otherwise, the Kruskal–Wallis test was applied. The significance level was set at *p* ≤ 0.05.

## 3. Results

### 3.1. M. fasciculata Geopropolis Cream Accelerates Wound Closure

**Figure 1:**
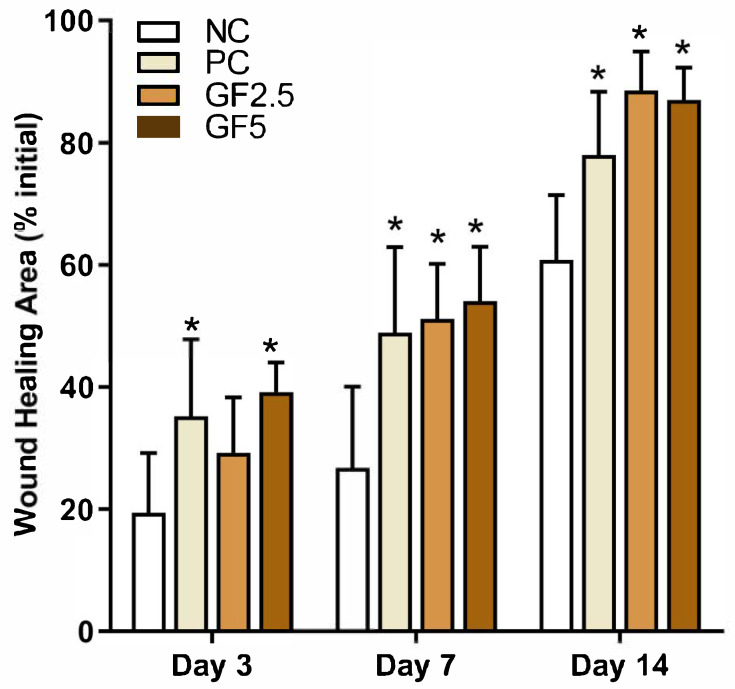
*M. fasciculata* geopropolis increases the percentage of repair and the speed of re-epithelialization in induced lesions in NOD mice. The wound areas were measured immediately after lesion induction and on the third, seventh, and fourteenth days, after which the percentage of repair was calculated. The values represent the means ± SDs of 12, 8, and 4 animals per group. * *p* ≤ 0.05 compared with the NC group on the same day (two-way ANOVA). NC: negative control group; PC: positive control group; GF2.5: 2.5% Geopropolis group; GF5: 5% Geopropolis group.

Initially, we evaluated the effects of the geopropolis formulations on the percentage of epithelial closure over time (Figure 1). On day 3, the PC and GF5 groups presented a greater percentage of epithelial closure than did the NP group. By day 7, the geopropolis-treated and PC groups presented a significant increase in the healed area relative to the control group. This trend of accelerated wound closure continued in the GF and PC groups until day 14. We found no significant differences in wound closure between the geopropolis-treated and PC-treated groups. These findings indicate that the geopropolis cream effectively accelerated wound closure in diabetic mice.

### 3.2. M. fasciculata Geopropolis Cream Alters Histopathological Parameters of Lesions

Histopathological analysis revealed that treatment with geopropolis cream led to significant changes in the evaluated parameters (Table 1). On days 3 and 7, all the groups exhibited moderate inflammatory infiltration, which was predominantly composed of polymorphonuclear cells. By day 14, mononuclear cells predominated, with a lower intensity of inflammatory infiltration in the geopropolis-treated groups, especially in the GF5 group.

On day 3, we observed no edema in the GP5 group, whereas the other groups presented moderate edema. On days 7 and 14, all groups maintained a similar pattern, with an absence or sparse presence of edema in the lesions.

We also analyzed the impact of treatment on the formation of new vessels. On day 3, we detected sparse angiogenesis in all the groups. However, by day 7, the NC and PC groups exhibited moderate angiogenesis, whereas the geopropolis-treated groups maintained the same sparse pattern observed on day 3. On day 14, this pattern persisted, with a moderate presence of new vessels only in the GP5 group. These findings suggest that the geopropolis cream did not significantly increase wound angiogenesis.

Additionally, on day 3, we observed a moderate presence of fibroblasts in the geopropolis-treated groups. In contrast, no fibroblasts were observed in the NC group, and a low intensity was observed in the PC group. By day 7, fibroblast presence intensified in the geopropolis-treated groups, with scarce and moderate presence in the NC and PC groups, respectively. On day 14, this difference remained, with moderate fibroblast proliferation in the geopropolis-treated groups and low proliferation in the other groups.

Because of this effect on fibroblast proliferation, by day 14, we observed moderate collagen deposition in the geopropolis-treated groups, whereas the CN and DFC groups presented low collagen deposition, similar to what was observed in all the groups on days 3 and 7. These findings suggest that the geopropolis cream may have a positive influence on wound collagen fiber synthesis.

### 3.3. M. fasciculata Geopropolis Cream Does Not Alter Serum Cytokine Production in Mice

**Figure 2:**
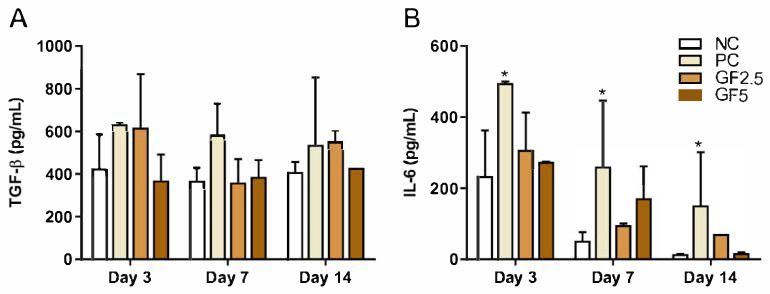
*M. fasciculata* geopropolis cream does not alter the production of (**A**) TGF-beta or (**B**) IL-6 in NOD mice. Quantification of the serum TGF-beta and IL-6 levels in the animals was performed on days 3, 7, and 14 of the experiment. Concentrations were determined via enzyme-linked immunosorbent assay (ELISA). The values represent the means ± SDs of four animals per group. * *p* ≤ 0.05 compared with the NC group on the same day (two-way ANOVA). NC: negative control group; PC: positive control group; GF2.5: 2.5% Geopropolis group; GF5: 5% Geopropolis group.

We analyzed the impact of treatment on the production of the cytokines TGF-beta and IL-6 (Figure 2). The TGF-beta levels remained similar among all the groups on days 3, 7, and 14, with no significant differences observed.

In contrast, IL-6 levels were consistently higher in the PC group than in the NC group on all analyzed days. However, we observed a progressive reduction in the IL-6 concentration over time in the PC group. Compared with those in the NC group, the IL-6 levels in the geopropanol-treated groups did not significantly differ.

### 3.4. β-Amyrin and Cycloartenol Bind Effectively to hMPO in Docking Analysis

The results of the molecular docking studies revealed the free binding energies (ΔGbind) between beta-amyrin and cycloartenol and human myeloperoxidase (hMPO). For hMPO, beta-amyrin and cycloartenol presented ΔGbind values of −9.406 and −8.430 kcal/mol, respectively. This result indicates the considerable affinity of *M. fasciculata* geopropolis extract compounds for hMPO.

**Figure 3:**
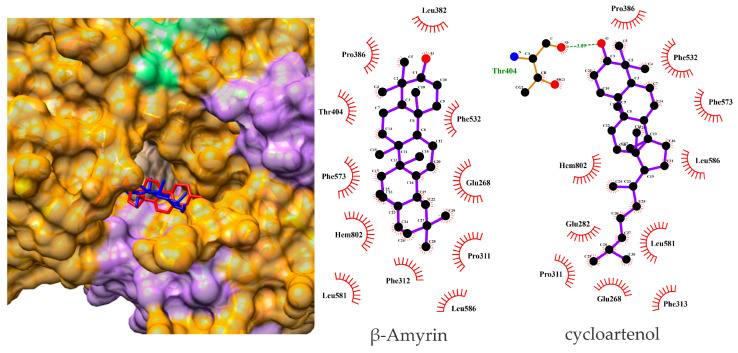
Surface representation of the docked position of beta-amyrin (in blue) and cycloartenol (in red) with the hMPO structure are represented and two-dimensional diagram of the interactions performed by these compounds with the amino acid residues and HEME group of the hMPO active site. Represented by LigPlot++.

Upon analyzing the complexes of beta-amyrin and cycloartenol with hMPO derived from molecular docking studies, it was observed that the compounds formed numerous interactions with the amino acid residues in the active sites of the target, with van der Waals interacting with Glu268, Pro311, Phe312, Phe313, Leu382, Pro386, Thr404, Phe532, Phe573, Leu581, and Leu586 and Heme group with beta-amyrin. The cycloartenol formed a hydrogen bond with Thr404 and van der Waals interacting with Glu268, Glu282, Pro311, Phe313, Pro386, Phe532, Phe573, Met577, Leu581, Leu586 and hMPO Heme group. The maximum RMSD observed between the conformation of the native ligand in the crystallographic structure and its conformation during redocking via our protocol was 1.02 Å, indicating that our protocol was effective in predicting interactions. The spatial conformations obtained by molecular docking of beta-amyrin and cycloartenol with the selected target are presented in Figure 3.

## 4. Discussion

The wound healing properties of *Apis mellifera* bee propolis have been confirmed for both acute [29] and chronic [30] lesions in both animal models and humans [31]. However, no evidence is available on whether *M. fasciculata* geopropolis exhibits these properties.

**Figure 4:**
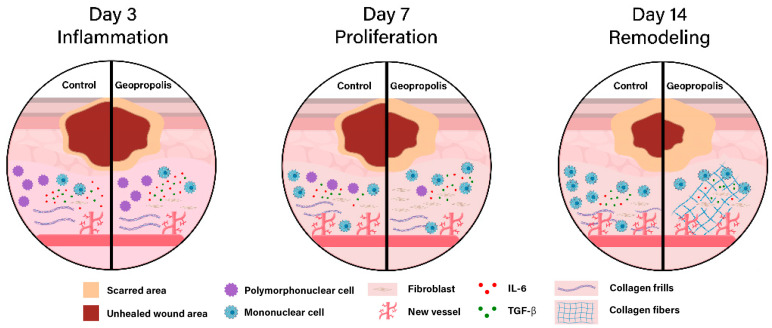
Proposed effect of *M. fasciculata* geopropolis cream on wound healing in diabetic NOD mice. Schematic representation of the inflammatory (day 3), proliferative (day 7), and remodeling (day 14) phases. In each circle, the left half shows the untreated control, and the right half shows the penicillin-treated wound. Geopropolis reduced the area of unhealed wounds and edema on day 3. On day 7, it increased the number of fibroblasts, supported collagen deposition, and maintained sparse angiogenesis, with TGF-beta present in the tissue. By day 14, the treated wounds exhibited marked contraction, organized collagen fibers, and minimal inflammatory cells.

In this study, we showed for the first time that topical treatment with *M. fasciculata* geopropolis cream accelerated wound healing in diabetic NOD mice. Our findings suggest that this effect is related to reduced inflammation and increased fibroblast proliferation and activity (Figure 4).

Although no prior study has examined the wound-healing activity of geopropolis from *M. fasciculata*, Sousa-Fontoura et al. (2020) [14] reported that 10% geopropolis cream from *M. subnitida* accelerated the healing and re-epithelialization of acute cutaneous wounds in rats. Despite differences in concentration, bee species, and lesion model, these findings are consistent with our findings, suggesting that geopropolis from distinct botanical and entomological origins may share wound-healing properties, particularly in promoting re-epithelialization and tissue repair.

In addition to accelerating wound closure, *M. fasciculata* geopropolis provides additional benefits that are critical for chronic wounds. Geopropolis from *M. fasciculata* has antibacterial activity, which is essential for preventing wound infections [12,32], a common complication in chronic lesions such as diabetic ulcers [2]. Furthermore, geopropolis exhibits potent antioxidant properties that neutralize free radicals [10,11,13], potentially reducing oxidative stress at the wound site and promoting a more favorable healing environment. Oxidative stress is a critical factor that can delay wound healing, especially in diabetic patients [33]. Moreover, *M. fasciculata* geopropolis has anti-inflammatory and immunomodulatory effects [13,34], which may help regulate the inflammatory response and promote efficient healing. These combined properties make *M. fasciculata* geopropolis a promising therapeutic option for treating chronic wounds, enhancing wound closure and the overall quality of regenerated tissue.

Histopathologic analysis revealed the cellular events linked to faster closure. Geopropolis treatment reduced the intensity of the inflammatory infiltrate during the remodeling phase on day 14. In acute lesions, this phase is characterized by reduced inflammatory infiltration, a greater presence of fibroblasts, organized collagen, and less angiogenic activity. In contrast, in chronic lesions such as diabetic ulcers, persistent inflammation from neuropathy and ischemia disrupts the normal healing phase, causing delays in the process [2,3]. The ability of the geopropolis cream to promote an efficient transition from the inflammatory phase to the remodeling phase is essential for proper healing. The reduced presence of inflammatory cells on day 14 indicates that geopropolis may help prevent progression to chronic inflammation, a major obstacle in wound healing for diabetic patients.

Although the serum IL-6 concentration decreased over time in all groups, the topical treatment with geopropolis did not alter its systemic concentration. IL-6 is a pleiotropic cytokine that orchestrates the transition from inflammation to tissue repair through both classic and trans-signaling pathways. It promotes leukocyte infiltration, keratinocyte and fibroblast proliferation, and endothelial activation and engages in an IL-6/TGF-beta feedback loop. This tightly regulated temporal expression is crucial for preventing chronic inflammation and fibrosis, underscoring the context-dependent role of IL-6 in cutaneous wound healing. Typically, plasma IL-6 peaks within 4–6 h after injury and decreases over the next 10 days [35]. Our data align with this kinetic pattern, suggesting that the cream exerts its effects locally without altering systemic cytokine profiles. However, profiling cytokine expression in wound tissue is necessary to confirm local immunomodulatory effects.

Chemical analysis revealed that this geopropolis sample contains beta-amyrin and cycloartenol as its major components [11]. Both triterpenoids are recognized for their anti-inflammatory properties [36], which may explain the observed efficiency of the geopropolis treatment of lesions. Beta-amyrin isolated from *Costus igneus* Nak. inhibits the activities of cyclooxygenase-2 (COX-2), myeloperoxidase (MPO), and nitric oxide synthase (NOS). In addition, it reduces the secretion of prostaglandin E2 (PGE2) and IL-6 as well as the activation of nuclear factor kappa B (NF-κB) in human peripheral blood mononuclear cells [37]. In an acute periodontitis model, this triterpenoid reduced tumor necrosis factor-alpha (TNF-alpha), MPO, and thiobarbituric acid reactive substances (TBARS) [38].

When tissue injury occurs, resident macrophages release various cytokines, including TNF-alpha. TNF-alpha acts on endothelial cells and induces the expression of leukocyte adhesion molecules such as the integrin ligands VCAM1 (vascular cell adhesion molecule-1) and ICAM-1 (intercellular adhesion molecule-1) [39]. Agents that block TNF-alpha, one of the main leukocyte-recruiting cytokines, reduce leukocyte adhesion and, consequently, the intensity of the inflammatory infiltrate and are among the most successful medications for treating chronic inflammatory diseases [40]. 

Ahumada et al. (1997) [41] reported that the cycloartenol fraction of *Crataegus monogyna* Jacq. inhibits leukocyte infiltration in the peritoneum of mice. The authors showed that cycloartenol inhibits phospholipase A2, disrupting the arachidonic acid cascade and reducing local inflammation. Furthermore, in an experimental colitis model, cycloartenyl ferulate attenuated both the clinical signs and symptoms of the disease and inhibited MPO activity [42]. Since this compound can be cleaved by pancreatic or intestinal enzymes into cycloartenol and ferulic acid [43], it is possible that cycloartenol alone may contribute to the anti-inflammatory effects observed.

To probe the molecular mechanism, we performed in silico docking of beta-amyrin and cycloartenol with human myeloperoxidase (hMPO). The negative free binding energy values reported by Lopes et al. (2022) [26] suggest that these interactions favor the formation of the ligand–receptor complex. Both ligands showed favorable binding energies (ΔG_bind ≈ –9.4 and –8.4 kcal mol^−1^, respectively) and occupied the catalytic pocket through van der Waals contacts and hydrogen bonding. Although these findings support a potential inhibitory role of MPO, functional validation through enzymatic assays is still needed to confirm its biological relevance.

Myeloperoxidase plays a crucial role in the early stages of the inflammatory response by producing reactive oxygen species (ROS), which are essential for the elimination of pathogens. However, excessive MPO activity can lead to increased oxidative stress, which may impair tissue regeneration and delay wound healing; thus, proper regulation of MPO activity is critical [44,45]. Controlled MPO activity helps reduce inflammatory infiltrates, facilitating the transition to subsequent phases of wound healing, such as granulation tissue formation and tissue remodeling [46,47].

Our molecular docking studies provide theoretical evidence that beta-amyrin and cycloartenol can act as potential MPO inhibitors. By modulating MPO activity, these compounds could help mitigate oxidative stress, creating a more favorable environment for tissue repair. Thus, beta-amyrin and cycloartenol have emerged as promising candidates for the development of therapeutic agents aimed at enhancing and accelerating the wound healing process. The ability of beta-amyrin and cycloartenol to inhibit myeloperoxidase activity is crucial for treating chronic lesions, as persistently activated neutrophils at the lesion site stimulate an increase in these proteases, compromising cell migration and promoting the destruction of protein matrices and growth factors necessary for repair [46].

Neutrophils are essential in the initial phases of healing but are detrimental if they persist in subsequent phases. Other studies have shown that in diabetic mouse lesions, the number of neutrophils decreases during the inflammatory phase of repair but remains stable during subsequent phases [48]. In our study, the reduction in the number of neutrophils after the inflammatory phase and the mononuclear profile of the infiltrate demonstrated the beneficial action of geopropolis. Additionally, by reducing injury caused by reactive oxygen species [10,11,13], *M. fasciculata* geopropolis may help decrease the inflammatory response. In parallel, cycloartenol-induced upregulation of the enzymes COL1A1 and COL1A3, which are responsible for collagen synthesis, and HAS2 and HAS3, which promote hyaluronic acid synthesis [49], may explain the increased fibroblast density and improved collagen organization observed in treated wounds. Trace elements, unsaturated fatty acids, steroids, and organic acids present in geopropolis [50] may further stimulate fibroblast activity.

Despite the positive effect on fibroblast proliferation and activity, topical treatment with geopropolis did not increase the serum levels of transforming growth factor-beta (TGF-beta), which is considered one of the most potent fibrogenic agents necessary for cell proliferation and extracellular matrix deposition and is deficient in diabetic ulcers [51]. *A. mellifera* propolis and some of its constituents stimulate the release of TGF-beta1 by human lymphocytes [52]. The absence of changes in the serum concentration of this cytokine may be related to the route of treatment used. Topical treatments tend not to produce systemic effects, reducing the risk of adverse effects.

Collectively, these findings indicate that *M. fasciculata* geopropolis cream accelerates diabetic wound healing by tempering early inflammation, inhibiting MPO-mediated oxidative injury, and enhancing fibroblast-driven collagen synthesis—all without altering systemic cytokine profiles.

## 5. Conclusions

Our findings demonstrate that the geopropolis of *M. fasciculata* cream accelerates epithelial closure in diabetic wounds in NOD mice. This effect is associated with both a reduction in inflammatory infiltration and a shift from polymorphonuclear to mononuclear cells during the later stages of healing. Additionally, the cream promotes increased fibroblast proliferation and collagen fiber production, which are crucial for effective wound healing. The increased presence of fibroblasts suggests a greater capacity for extracellular matrix synthesis, whereas the increased number of collagen fibers indicates improved tissue organization and scar strength.

These results highlight the potential of the geopropolis of *M. fasciculata* as a therapeutic alternative for treating diabetic ulcers. In this experimental model, the cream reduces healing time and improves tissue quality, potentially minimizing complications such as infections and amputations. Molecular docking analysis revealed that the major compounds in this geopropolis—beta-amyrin and cycloartenol—have the potential to inhibit human myeloperoxidase (hMPO), which may contribute to the observed effects.

Future studies should investigate the molecular mechanisms underlying its activity and assess the therapeutic potential of its isolated bioactive compounds. Overall, our findings provide preliminary preclinical evidence that *M. fasciculata* geopropolis cream may enhance wound healing under diabetic conditions. However, clinical trials are necessary to confirm its efficacy and safety for human therapeutic use.

## Figures and Tables

**Table 1:** Effect of the geopropolis cream treatment on the histopathological parameters of lesions in NOD mice.

Parameters Analyzed	Day 3				Day 7				Day 14			
	NC	PC	GF2.5	GF5	NC	PC	GF2.5	GF5	NC	PC	GF2.5	GF5
Inflammatory Infiltrate	++	++	++	++	++	++	++	++	+++	+++	+	++
Edema	++	++	++	-	+	+	+	+	+	+	-	+
Angiogenesis	+	+	+	+	++	++	+	+	++	++	+	++
Fibroblasts	-	+	++	++	+	++	+++	+++	+	+	++	++
Collagen Fibers	+	+	+	+	+	+	+	+	+	+	++	++

NC: negative control group; PC: positive control group; GF2.5: 2.5% Geopropolis group; GF5: 5% Geopropolis group.

## Data Availability

The original contributions presented in this study are included in the article. Further inquiries can be directed to the corresponding author.
